# ASO Author Reflections: Bang for Your Buck—Implications of the Cost-Effectiveness of Watchful Waiting Versus Radical Surgery for Locally Advanced Rectal Cancer After Successful Neoadjuvant Chemoradiation

**DOI:** 10.1245/s10434-021-10616-8

**Published:** 2021-09-14

**Authors:** Christina Liu Cui, William Yu Luo, Bard Clifford Cosman, Samuel Eisenstein, Daniel Simpson, Sonia Ramamoorthy, James Murphy, Nicole E. Lopez

**Affiliations:** 1 Department of Surgery, Duke University Hospital, Durham, NC USA; 2 School of Medicine, University of California, San Diego, La Jolla, CA USA; 3 Department of Surgery, University of North Carolina Hospitals, Chapel Hill, NC USA; 4 Department of Surgery, Division of Colon and Rectal Surgery, University of California, San Diego Health Systems, La Jolla, CA USA; 5 Veterans Affairs San Diego Medical Center, San Diego, CA USA; 6 Department of Radiation Medicine and Applied Science, University of California, San Diego, La Jolla, CA USA

## Past

Historically, treating rectal cancer has been a challenging endeavor. We have optimized local recurrence with preoperative radiation and total mesorectal excision (TME). Unfortunately, surgical resection, which remains the standard of care, can have substantial negative effects on patients’ quality of life.

In 2004, Habr-Gama reported excellent outcomes using a nonoperative (“watch and wait” [WW]) approach for patients with rectal cancer and complete clinical response (cCR) after chemoradiation.^1^ Since that time, multiple observational studies have confirmed this. In 2017, a meta-analysis of 876 patients with rectal cancer and cCR confirmed no difference in non-regrowth recurrence, cancer-specific mortality, disease-free survival or overall survival among patients treated with WW versus surgery.^2^

## Present

In an era where health care is dominated by soaring expenditures and outcomes disproportionate to increasing costs, and where the importance of patient-centered care is gaining recognition, we sought to perform a more global assessment of the utility of WW.^3,4^ We used Markov modeling to show that WW was less expensive and offered greater health utility compared with TME.^5^

## Future

Our study cannot address the clinical efficacy of WW; randomized trials are necessary. However, our results highlight the need to consider that societies and patients alike will be inclined to pursue WW unless controlled trials indicate it is drastically inferior—an unlikely circumstance given current observational data. As such, we must implement standards to assist physicians and institutions in properly selecting and monitoring patients undergoing WW. Our findings also emphasize the role of patient centered outcomes in assessing therapy; patient centered outcomes also should be standardized and could be addressed similarly to oncologic outcomes. Finally, we should commit to continuing to search for therapies that increase the rate of cCR as this indicates excellent outcomes, regardless of further management. Ideally, the end-goal is equitable distribution of well-informed, carefully practiced, patient-oriented care.

## References

[1] Habr-Gama A, Perez RO, Nadalin W (2004). Operative versus nonoperative treatment for stage 0 distal rectal cancer following chemoradiation therapy: long-term results. Ann Surg..

[2] Dossa F, Chesney TR, Acuna SA, Baxter NN (2017). A watch-and-wait approach for locally advanced rectal cancer after a clinical complete response following neoadjuvant chemoradiation: a systematic review and meta-analysis. Lancet Gastroenterol Hepatol..

[3] OECD Health Statistics 2020 (2020).

[4] Selby JV, Beal AC, Frank L (2012). The Patient-Centered Outcomes Research Institute (PCORI) national priorities for research and initial research agenda. JAMA.

[5] Cui CL, Luo WY, Cosman BC, et al. Cost-effectiveness of watch and wait versus resection in rectal cancer patients with complete clinical response to neoadjuvant chemoradiation. *Ann Surg Oncol*. 2021. 10.1245/s10434-021-10576-z. 10.1245/s10434-021-10576-z PMC8810473 34529175

